# Effects of life-sustaining treatment plans on healthcare expenditure and healthcare utilization

**DOI:** 10.1186/s12913-023-10235-x

**Published:** 2023-11-10

**Authors:** Wonjeong Jeong, Selin Kim, Hyunkyu Kim, Eun-Cheol Park, Sung-In Jang

**Affiliations:** 1 Cancer Knowledge & Information Center, National Cancer Control Institute, National Cancer Center, Goyang, Republic of Korea; 2 Review and Assessment Research Department, Health Insurance Review & Assessment Service, Wonju, Republic of Korea; 3 Department of Psychiatry, Yonsei University College of Medicine, Seoul, Republic of Korea; 4 Department of Preventive Medicine & Institute of Health Services Research, Yonsei University College of Medicine, 50 Yonsei-ro, Seodaemun-gu, Seoul, 03722 Republic of Korea

**Keywords:** Life support care, Withholding treatment, Terminal care, Health expenditures, Health utilization

## Abstract

**Purpose:**

To develop an ethical and cultural infrastructure for Life-Sustaining Treatment (LST) plan, it is crucial to carefully analyze its impact and ensure that healthcare utilization is maintained at an appropriate level, avoiding excessive medical interventions. This study aims to investigate the effects of LST decisions on both healthcare expenditure and utilization.

**Methods:**

This cohort study utilized claims data from the National Health Insurance Service, encompassing all medical claims in South Korea. We included individuals who had planned to withdraw or withhold their LST between January and December 2018, identified by claim code IA71, IA72, IA73. We followed a total of 28,295 participants with documented LST plan who were deceased by June 2020. Participants were categorized into LST withdrawal / withholding and LST continuation groups. The dependent variables were healthcare expenditure and utilization. We construct a generalized linear model to analyze the association between these variables.

**Results:**

Out of the 28,295 participants, 24,436 (86.4%) chose to withdraw or withhold LST, while the rest opted for its continuation. Compared to the LST continuation group, those who chose to withdraw or withhold LST had 0.91 times lower odds for total cost. Additionally, they experienced 0.91 times fewer hospitalization days and 0.92 times fewer outpatient visits than those in the LST continuation group.

**Conclusion:**

Healthcare expenditure and utilization deceased among those choosing to withdraw or withhold LST compared to those continuing it. These findings underscore the significance of patients actively participating in decision regarding their treatment to ensure appropriate levels of medical intervention for LST. Furthermore, they emphasize the critical role of proper education and the establishment of a cultural framework for LST plans.

**Supplementary Information:**

The online version contains supplementary material available at 10.1186/s12913-023-10235-x.

## Introduction

Population aging is a major driver of the demand for healthcare and, thus, of the annual growth in national health spending [1, 2]. Korea, presently an aging society, is expected to become a post-aged society in 2025, when 20% of its total population will be composed of older adults aged 65 years and older [3]. The development of medical technology can not only improve health but also sustain life through proper treatment. However, such technology may also only prolong the process of death, where recovery is no longer feasible [4]. Moreover, patients and their family often report the cost of care as a major financial burden, with many ending up spending most or all of their savings on medical expenses [5]. Considering the changes in the population structure, such as low birth rates and aging, the efficient use of resources to cope with the changes in medical use grows increasingly salient.

As death approaches, patients are faced with higher pre-death medical expenditure, which is due to the development of medical technology that has increased the number of services, or due to the continuous services to patients provided by medical institutions [6]. In Korea, the average monthly medical expenditure, which is continuously increasing, was KRW 1,329,000 a year before death and KRW 2,417,000 shortly before death in 2015; their corresponding figures in 2005 were KRW 391,000 and KRW 909,000, respectively [6, 7]. Medical expenses for extending life tend to be lower in the period of healthy life gained through treatment [8]. Meanwhile, medical expenses for life-sustaining treatment (LST) do not significantly affect quality of life [6]. Therefore, the right to self-determination of LST for patients has become increasingly important.

The rights of patients to refuse various forms of medical care have been discussed with regard to end-of-life treatment [9]. The Korean government has sought to expand the foundation of the LST system, increasing the relevant budget for 2019 by 102.6% compared with 2018 [10]. The Law on the Hospice and Palliative Care and the Determination of Life-Sustaining Treatment for Terminally Ill Patients (Act No. 14,013) (henceforth, Determination of Life-Sustaining Treatment Act), which allowed terminally ill patients to opt out of LST [11], was enacted in January 2016 and came into effect in February 2018 [4]. However, the research on the actual efficacy of LST plans remains insufficient. Living a long life is important, but the quality of life is also important. For this reason, the demand for LST planning is increasing, which is expected to give self-determination to treatment and transform treatment culture and methods.

Therefore, to develop an ethical and cultural framework for LST planning, it is crucial to avoid excessive medical intervention and ensure that healthcare utilization remains at an appropriate level. We hypothesized that withdrawing or withholding LST among patients who choose to plan for it could reduce unnecessary medical care and maintain an appropriate level of medical intensity. Therefore, this study aims to evaluate the impact of LST decisions on healthcare expenditure and utilization, with the intent of establishing ethical and cultural guidelines for LST planning. LST planning empowers patients to make informed decision about their treatment, safeguarding their legal rights, as well as their dignity and values as human beings [4]. Consequently, this study holds significant potential to assist those contemplating LST planning, and could serve as a cornerstone in developing an ethical and cultural framework for LST.

## Methods

### Data and study participants

This study was conducted using the claims data collected by the National Health Insurance Service (NHIS) of South Korea between January 2018 to June 2020. The data included all medical claims of the South Korean population. The NHIS follow up with the patients, record their clinical and provisions’ characteristics over time, and inform them about the development of healthcare policies [12]. This database included unique de-identified patients’ numbers to mask personally identifiable information [12, 13]. We compiled the data for the sole purpose of providing public health researchers and policy makers with representative and useful information on Korean citizens’ use of health insurance and health examinations.

**Fig. 1:**
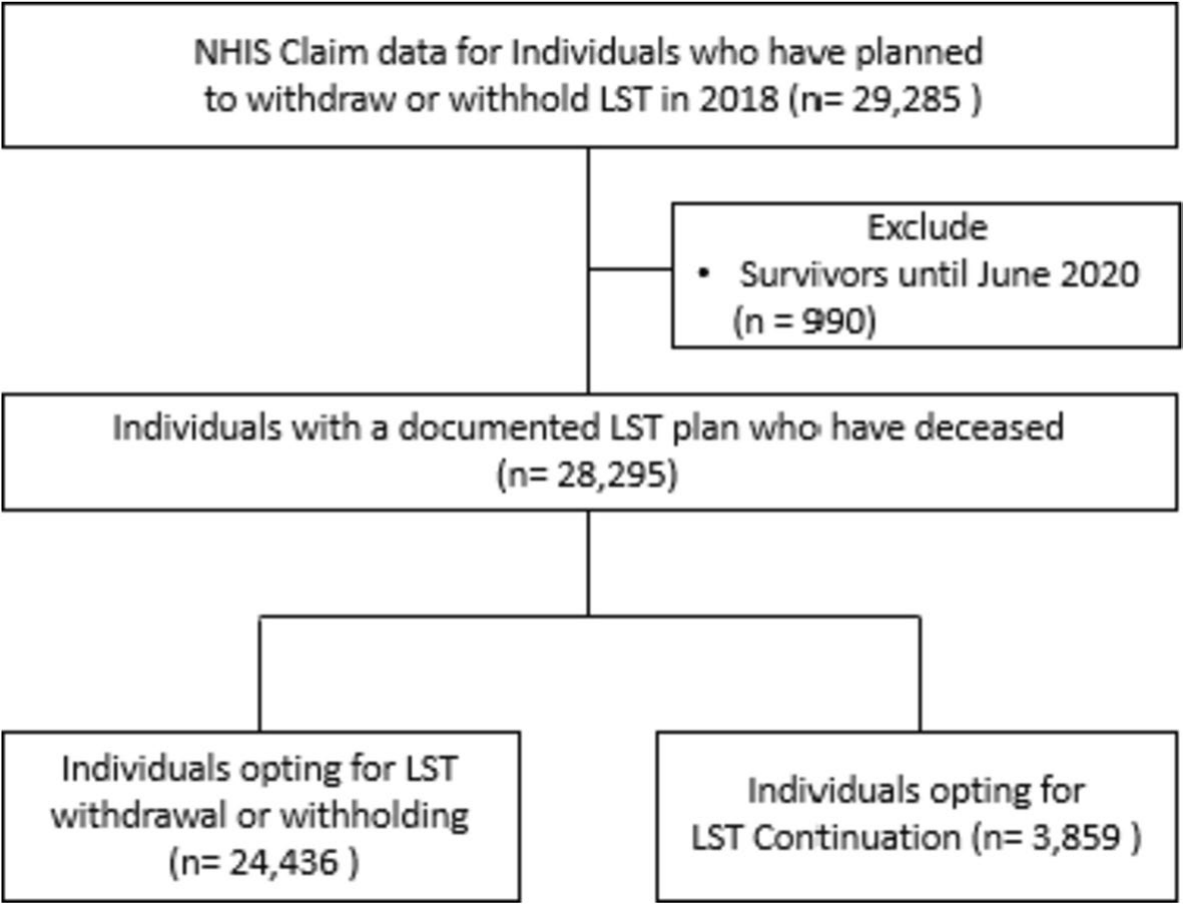
Flowchart of the participants selection. NHIS: National Health Insurance Service; LST: Life-Sustaining Treatment

From this data, 29,285 individuals who have planned to withdraw or withhold their LST (claims codes: IA71, IA72, IA73) between January and December 2018 were included in our analysis. To ensure homogeneity in participants characteristics, 990 individuals who survived during the follow-up period (January 2018 to June 2020) were excluded. Consequently, a total of 28,295 participants who had documented their LST plans and deceased were included in this study. Among them, 24,436 had opted for LST withdrawal or withholding, while the remaining 3,859 had opted for LST continuation (Fig. 1). This study adhered to the tenets of the Declaration of Helsinki and was based on the routinely collected administrative and claims data. We obtained the data with permission from NHIS (NHIS-2021-1-155). This study was reviewed and approved by the Institutional Review Board (IRB number: Y-2020-0193).

### Variables

The dependent variables in this study were healthcare expenditure and healthcare utilization calculated from the date when they planned LST to their death date. The former includes the total, hospitalization, outpatient, and medication costs, whereas the latter pertains to the days of hospitalization and outpatient visit. Health expenditure in South Korea encompasses the total valid cost incurred during treatment, which includes both the patient’s copayment and the insurance benefits paid by NHIS [14]. South Korea operates a compulsory social insurance system, NHIS, covering approximately 97% of the population. Most of the remaining 3% of the populations is covered by medical aid, which is funded by both the central and local government, allowing beneficiaries of medical aid to make relatively lower copayments [15]. All hospitals and clinics in South Korea are required to submit medical records of NHIS-covered patients, which include diagnosis and operation codes, to the Health Insurance Review & Assessment Service [16]. This process is essential to secure reimbursement for any healthcare service provided. Healthcare expenditure was expressed in KRW (USD 1 = KRW 1,129.40 on March 22, 2021). The primary independent variable was LST decision, categorized into LST withdrawal / withholding and LST continuation groups, defined by NHIS claims codes IA74. Additionally, our analyses included the main disease, sex, age, insurance premium, and medical institution.

### Statistical analysis

We presented the sample’s general characteristics as frequencies and percentages. To examine the distribution of the study population’s general characteristics in terms of healthcare expenditure and utilization, we conducted a *t*-test and analysis of variance. The association between LST decision and healthcare expenditure and utilization was assessed using a generalized linear model (GLM). For the analysis of the association between LST decision and healthcare expenditure, we employed the log link function and utilized the Gamma distribution. In the case of healthcare utilization, we used log for the link function and employed the negative binomial distribution. Differences with a *p*-value < 0.05 were considered statistically significant. All data analyses were conducted using SAS Enterprise 7.1 (SAS Institute Inc., Cary, NC, USA).

## Results

Table 1 presents the general characteristics of the study population those who have planned LST. A large majority (86.4%) opted for LST withdrawal or withholding, while the rest (13.6%) chose to continue their LST. Among those who had planned their LST, more than half (56.9%) had cancer. Table 2 shows the general characteristics of the study population’s healthcare expenditure and utilization. The mean total cost of those who opted for withdrawal or withholding LST was ₩15,966,911, and ₩16,788,299 for the LST continuation group. The mean total cost was ₩14,493,796, ₩17,731,190, and ₩17,611,346 for those who had cancer, circulatory system issues, and respiratory system issues, respectively. The mean number of hospitalizations of the LST withdrawal or withholding group was 81.77 days, compared to 87.85 days for the LST continuation group. The mean number of hospitalizations was 79.10 days, 66.43 days, and 96.50 days for the those who had cancer, circulatory system issues, and respiratory system issues, respectively.

**Table 1:** General characteristics of the study population, categorized by the decision to opt for Life-Sustaining Treatment (LST) withdrawal / withholding or not, among those who have planned LST

Variables	Total		Life-Sustaining Treatment decision			
			LST withdrawal or withholding		LST Continuation	
	N	(%)	N	(%)	N	(%)
Total	28,295	(100.0)	24,436	(86.4)	3,859	(13.6)
Main illness						
Cancer	16,092	(56.9)	13,227	(82.2)	2,865	(17.8)
Circulatory system	2,571	(9.1)	2,441	(94.9)	130	(5.1)
Respiratory system	3,829	(13.5)	3,515	(91.8)	314	(8.2)
Others	5,803	(20.5)	5,253	(90.5)	550	(9.5)
Sex						
Male	17,057	(60.3)	14,664	(86.0)	2,393	(14.0)
Female	11,238	(39.7)	9,772	(87.0)	1,466	(13.0)
Age (years)						
< 50	2,010	(7.1)	1,705	(84.8)	305	(15.2)
50–60	3,770	(13.3)	3,166	(84.0)	604	(16.0)
60–70	5,986	(21.2)	5,087	(85.0)	899	(15.0)
70–80	8,291	(29.3)	7,152	(86.3)	1,139	(13.7)
≥ 80	8,238	(29.1)	7,326	(88.9)	912	(11.1)
Insurance premium (percentile)						
Medical Aid	2,455	(8.7)	2,123	(86.5)	332	(13.5)
1 (Low)	3,951	(14.0)	3,378	(85.5)	573	(14.5)
2	3,179	(11.2)	2,735	(86.0)	444	(14.0)
3	4,114	(14.5)	3,538	(86.0)	576	(14.0)
4	5,490	(19.4)	4,763	(86.8)	727	(13.2)
5 (High)	9,106	(32.2)	7,899	(86.7)	1,207	(13.3)
Medical institution						
Tertiary hospital	18,164	(64.2)	15,345	(84.5)	2,819	(15.5)
General hospital	10,091	(35.7)	9,056	(89.7)	1,035	(10.3)
Others	40	(0.1)	35	(87.5)	5	(12.5)

**Table 2:** Results of Mean and SD of the study populations’ healthcare expenditure and utilization

Variables	Healthcare expenditure				Healthcare utilization	
	Total cost	Hospitalization cost	Outpatient cost	Medication cost	Days of hospitalization	Outpatient visit
	Mean SD	Mean SD	Mean SD	Mean SD	Mean SD	Mean SD
Life-Sustaining Treatment						
Withdrawal/withholding	15,996,911 ± 20,519,104	15,688,035 ± 20,055,408	215,799 ± 1,751,702	93,071 ± 1,396,000	81.77 ± 88.5	27.59 ± 28.5
Continuation	16,788,299 ± 19,485,984	15,848,890 ± 18,494,712	669,505 ± 2,883,627	269,903 ± 2,472,397	87.85 ± 87.3	31.88 ± 29.1
Main illness						
Cancer	14,493,976 ± 18,195,044	14,027,260 ± 17,413,304	331,695 ± 2,254,810	135,021 ± 2,013,641	79.10 ± 70.2	30.99 ± 27.3
Circulatory system	17,731,190 ± 22,132,820	17,515,365 ± 22,041,197	127,485 ± 533,936	88,340 ± 624,811	66.43 ± 100.9	24.08 ± 29.8
Respiratory system	17,611,346 ± 21,034,053	17,370,535 ± 20,917,873	126,531 ± 796,414	114,280 ± 743,492	96.50 ± 116.5	22.96 ± 29.4
Others	18,857,276 ± 24,115,078	18,480,656 ± 23,617,353	294,160 ± 1,972,766	82,435 ± 710,373	90.31 ± 103.1	25.63 ± 30.3
Sex						
Male	15,693,583 ± 19,611,486	15,323,034 ± 19,161,391	264,742 ± 1,918,940	105,798 ± 1,353,096	77.27 ± 80.7	28.80 ± 29.1
Female	16,729,055 ± 21,486,459	16,297,268 ± 20,837,820	297,311 ± 1,999,627	134,476 ± 1,888,443	90.69 ± 98.4	27.23 ± 27.9
Age (year)						
<50	20,637,659 ± 29,348,504	20,227,316 ± 28,731,898	247,502 ± 1,748,048	162,842 ± 3,225,514	93.97 ± 86.1	24.23 ± 23.6
50–60	17,105,452 ± 22,906,943	16,636,435 ± 22,270,055	356,335 ± 2,810,268	112,642 ± 1,053,742	87.39 ± 80.0	26.68 ± 26.5
60–70	16,078,047 ± 20,157,057	15,688,709 ± 19,493,528	295,504 ± 1,912,051	93,834 ± 744,410	84.14 ± 82.1	30.63 ± 29.6
70–80	15,870,555 ± 18,762,769	15,455,003 ± 18,213,471	286,808 ± 1,835,585	128,745 ± 1,981,056	78.78 ± 83.5	31.38 ± 29.7
≥80	14,796,231 ± 17,892,508	14,455,862 ± 17,553,922	226,902 ± 1,633,444	113,468 ± 1,121,482	80.37 ± 100.6	24.83 ± 28.4
Insurance premium (percentile)						
Medical Aid	16,173,168 ± 19,054,179	15,745,377 ± 18,373,753	286,392 ± 1,812,605	141,339 ± 1,328,502	96.37 ± 100.5	25.63 ± 28.7
1 (Low)	15,427,726 ± 18,483,861	15,057,700 ± 18,098,313	274,396 ± 1,641,667	95,630 ± 632,639	79.17 ± 85.9	27.88 ± 29.1
2	16,057,963 ± 19,767,465	15,595,409 ± 18,918,198	331,695 ± 3,055,709	130,859 ± 1,682,188	79.72 ± 84.9	27.67 ± 28.9
3	15,821,402 ± 20,858,617	15,515,271 ± 20,536,469	232,075 ± 1,748,308	74,056 ± 561,501	78.51 ± 81.9	27.00 ± 27.1
4	15,912,201 ± 20,156,981	15,547,905 ± 19,697,226	267,589 ± 1,594,777	96,707 ± 945,211	80.64 ± 82.6	28.86 ± 28.2
5 (High)	16,640,786 ± 21,599,854	16,209,114 ± 21,011,548	284,579 ± 1,898,319	147,092 ± 2,347,578	84.42 ± 92.8	29.30 ± 29.2
Medical Institution						
Tertiary Hospital	16,808,025 ± 21,118,173	16,382,059 ± 20,562,982	304,021 ± 2,166,254	121,945 ± 1,796,431	82.00 ± 86.0	29.67 ± 29.3
General Hospital	14,861,278 ± 18,927,750	14,521,460 ± 18,438,583	230,942 ± 1,492,426	108,862 ± 1,121,453	83.52 ± 92.0	25.53 ± 27.3
Others	10,510,920 ± 18,930,941	10,348,234 ± 18,924,856	105,398 ± 224,507	57,289 ± 113,600	123.55 ± 166.3	20.38 ± 22.5

Table 3 presents the GLM results for LST decisions and their impact on the study population’s health expenditure. Compared with the subjects in the LST continuation group, the odds of total cost were 0.91 times lower for those who chose to withdraw or withhold LST. Similarly, hospitalization cost were 0.94 times lower, outpatient cost were 0.67 times lower, and medication cost were 0.88 times lower among this group. Additionally, the odds of total cost were 1.30 times higher for those with circulatory system disease and 1.34 times higher for those with respiratory system disease compared to those with cancer, in terms of total cost. We also performed subgroup analyses on healthcare expenditure according to LST decision (S-Table 1). Participants with cancer who withdrew or withheld the LST had 0.92 times lower total cost than those with LST continuation. Among men who withdrew or withheld LST, the total cost was 0.90 times lower than those with LST continuation. As age increased, LST withdrawal or withholding group showed lower odds for total cost.

**Table 3:** Modeling results for Life-Sustaining Treatment decision with respect to healthcare expenditure

Variables	Total cost		Hospitalization cost		Outpatient cost		Medication cost	
	EXP (ß)	95% CI	EXP (ß)	95% CI	EXP (ß)	95% CI	EXP (ß)	95% CI
Life-sustaining treatment								
Withdrawal /withholding	0.91	(0.89-0.94)	0.94	(0.91-0.97)	0.67	(0.62-0.73)	0.88	(0.79-0.98)
Continuation	1.00		1.00		1.00		1.00	
Main illness								
Cancer	1.00		1.00		1.00		1.00	
Circulatory system	1.30	(1.25-.35)	1.32	(1.27-1.37)	0.53	(0.46-0.61)	1.20	(0.98-1.48)
Respiratory system	1.34	(1.30-1.39)	1.36	(1.32-1.41)	0.53	(0.47-0.60)	1.19	(1.02-1.39)
Others	1.35	(1.32-1.39)	1.36	(1.33-1.40)	1.14	(1.03-1.25)	1.00	(0.87-1.16)
Sex								
Male	1.00		1.00		1.00		1.00	
Female	1.08	(1.06-1.11)	1.08	(1.06-1.10)	1.12	(1.04-1.21)	1.19	(1.08-1.32)
Age (years)								
< 50	1.00		1.00		1.00		1.00	
50–60	0.86	(0.82-0.91)	0.86	(0.82-0.90)	1.35	(1.13-1.61)	0.64	(0.49-0.83)
60–70	0.81	(0.77-0.85)	0.80	(0.77-0.84)	1.18	(1.01-1.40)	0.46	(0.36-0.59)
70–80	0.76	(0.72-0.79)	0.75	(0.72-0.79)	1.18	(1.00-1.38)	0.55	(0.43-0.70)
≥ 80	0.67	(0.64-0.70)	0.66	(0.63-0.70)	0.93	(0.79-1.09)	0.44	(0.34-0.55)
Insurance								
Medical aid	1.03	(0.98-1.08)	1.03	(0.98-1.08)	1.09	(0.94-1.28)	1.28	(1.04-1.57)
1 (Low)	1.00		1.00		1.00		1.00	
2	1.04	(1.00-1.09)	1.03	(0.99-1.08)	1.18	(1.02-1.36)	1.62	(1.32-1.98)
3	1.02	(0.98-1.06)	1.03	(0.99-1.07)	0.89	(0.78-1.02)	0.82	(0.68-0.99)
4	1.04	(1.00-1.08)	1.04	(1.00-1.08)	0.99	(0.87-1.12)	0.99	(0.84-1.18)
5 (High)	1.10	(1.06-1.14)	1.10	(1.06-1.14)	1.08	(0.96-1.21)	1.37	(1.17-1.60)
Medical institution								
Tertiary hospital	1.00		1.00		1.00		1.00	
General hospital	0.90	(0.88-0.92)	0.90	(0.88-0.92)	0.84	(0.77-0.90)	0.91	(0.82-1.01)
Others	0.61	(0.46-0.81)	0.61	(0.46-0.81)	0.30	(0.13-0.67)	0.14	(0.07-0.28)

The GLM results for the LST decision on healthcare utilization are shown in Table 4. Compared with the LST continuation group, the odds of hospitalization days were 0.91 times lower, and outpatient visit were 0.91 times lower for those who opted to withdraw or withhold LST. In the subgroup analysis of healthcare utilization according to LST decision (S-Table 2), those in the LST withdrawal or withholding group with cancer had 0.96 times lower for hospitalization and 0.90 times lower for outpatient visit. Those in the LST withdrawal or withholding group who were men also had 0.92 times lower hospitalization and outpatient visits. As the age increased, those in the LST withdrawal or withholding group had lower odds for hospitalization compared with the LST continuation group.

**Table 4:** Modeling results for Life-Sustaining Treatment decision with respect to healthcare utilization

Variables	Days of hospitalization		Outpatient visit	
	EXP (ß)	95% CI	EXP (ß)	95% CI
Life-sustaining treatment				
Withdrawal/withholding	0.91	(0.88-0.94)	0.92	(0.89-0.95)
Continuation	1.00		1.00	
Main illness				
Cancer	1.00		1.00	
Circulatory system	0.87	(0.83-0.90)	0.79	(0.76-0.83)
Respiratory system	1.32	(1.27-1.36)	0.75	(0.72-0.78)
Others	1.17	(1.13-1.20)	0.85	(0.82-0.88)
Sex				
Male	1.00		1.00	
Female	1.20	(1.17-1.22)	0.98	(0.95-1.00)
Age (years)				
< 50	1.00		1.00	
50–60	0.95	(0.90-1.00)	1.10	(1.04-1.16)
60–70	0.91	(0.86-0.95)	1.28	(1.22-1.34)
70–80	0.82	(0.78-0.85)	1.34	(1.28-1.41)
≥ 80	0.78	(0.75-0.82)	1.13	(1.07-1.18)
Insurance				
Medical aid	1.19	(1.14-1.25)	0.95	(0.90-0.99)
1 (Low)	1.00		1.00	
2	1.00	(0.96-1.04)	0.99	(0.95-1.04)
3	0.99	(0.95-1.03)	0.95	(0.91-0.99)
4	1.02	(0.98-1.06)	1.01	(0.98-1.06)
5 (High)	1.08	(1.05-1.12)	1.05	(1.01-1.09)
Medical institution				
Tertiary hospital	1.00		1.00	
General hospital	1.02	(0.99-1.04)	0.88	(0.86-0.91)
Others	1.38	(1.03-1.85)	0.75	(0.55-1.01)

## Discussion

Given the paramount significance of the quality of life in the context of medical interventions aimed at its extension, it becomes crucial to acknowledge patient’s rights to decline various forms of medical care [9]. In light of this, our study delves into the impact of LST decisions on healthcare expenditure and utilization among South Korean population. This research not only holds substantial potential in adding individuals in their considerations of LST planning but also lays the foundation for an ethical and cultural framework surrounding LST. Our findings indicate that, when compared to subjects in the LST continuation group, those who chose to withdraw or withhold LST exhibited lower odds in terms of healthcare expenditure, days of hospitalization, and outpatients visit.

Inappropriately aggressive treatment near the end of life may lead to higher resource utilization, increased cost, and decreased quality of life [17]. Indeed, aggressiveness of care near the end of life has not been associated with increased survival [17, 18]. Planning ahead and discussing one’s desires with the family is important because when the disease worsens, one may be unable to make decisions [19, 20]. In Korea, only 1.2% of individuals decide their LST plan by themselves; almost all of the decisions are done when the death is imminent or by members of the family [21]. Given the importance of advanced interest in LST planning, the ethical and cultural infrastructure surrounding this issue must be established.

South Korea’s LST system empowers patients to proactively plan the withdrawal of LST, providing them with the autonomy to make decisions. This approach ensures that the patient’s values are respected, even in cases where they are unable to express their intentions [22]. When a LST plan is in place, the decision to withdraw or continue LST can be made when the patient is determined to be the process of dying (Supplementary Fig. 1). During this process, if the patient is able to communicate, their intentions are reconfirmed towards the end of the life. If the patient is unable to communicate, two attending physicians follow a protocol to verify the legal validity of the written LST plan [22]. This study exclusively focuses on patient with documented LST plans. Those whose intention related to LST were not expressed in advance, including those who planned and then withdrew their plan, were not considered in this study due to this specific inclusion criteria.

LST plans should be in the form of written instructions to ensure that the individual’s wishes are clearly laid out and can be legally honored [19]. As the importance of self-determination in treatment increases, showing the effects of LST planning is necessary, to help guide, support, and protect physicians when making decisions on LST [23]. In other words, written LST plans protect both the patient and the physician. However, older adults tend to avoid making decisions on LST or recognize the discontinuation of LST as neglect [21]. To provide information and enhance awareness regarding LST, NHIS launched an education program for both the public and physicians in 2018 [24], thereby recognizing LST as an important issue in Korea and fostering a patient-centered treatment culture. An important finding from our study is that opting to withdraw or withhold LST led to reduced healthcare expenditure and utilization, compared to the LST continuation group. This finding serves as compelling evidence in favor of investing in LST education and the establishment of a culturally relevant treatment infrastructure.

Our study had several limitations. First, we could not distinguish between those who withdrew or withheld LST, although both are widely regarded as ethically equivalent in medical guidelines and ethics literature [25]. Second, it was not possible to standardize the follow-up time for subjects who underwent LST compared to those who did not. However, considering that the subjects with LST plans were individuals in the terminal stages of life, there may not be a significant discrepancy. Additionally, for the sake of homogeneity, we exclusively focused on individuals who had passed away. Since we only included those who have deceased before June 2020, there might be no significant difference in the follow-up period. The observed difference in mean costs also suggests it might be attributed to an escalation in unnecessary healthcare utilization. Third, if each individual still has an LST, there may be a possibility that doctors can maintain quality of life while extending their lifespan sufficiently. Lastly, owing to the lack of data, potential confounding variables, such as smoking status, drinking, and physical activity, could not be included. Moreover, we were unable to analyze information about the cause of death due to privacy concerns.

Despite these limitations, our study makes a significant contribution to literature for its use of national cohort data. Moreover, as the Determination of Life-Sustaining Treatment Act came into effect in February 2018 [4], research on LST planning is in its infancy. Therefore, this study could serve as a valuable reference for LST in Korea. Lastly, our findings could provide evidence for halting the practice of inappropriate LST, which only adds a burden to both patients and their family.

## Conclusions

Our values and choices play a significant role in shaping our lives. Decision-making in matters of LST should be a collaborative effort involving clinicians, patients, and their families. The enactment of Act on Decision on LST has brought about improvements in clinical practice, yet challenges persist for both patients and providers in its implementation. This study delves into the impact of LST decisions on healthcare expenditure and utilization among South Korean population. It not only encourages individuals to consider LST planning but also lays the foundation for an ethical and cultural framework surrounding LST. By emphasizing the importance of patients making their own decisions about LST, this study offers support to those contemplating their LST plan. Furthermore, it contributes to the broader discourse on LST, fostering greater awareness and openness in discussing LST planning among the public. Our study provides valuable insights for both official policy and current healthcare practices, offering potential benefits for future LST research [26]. The need for improved end-of-life regulatory frameworks and LST practice underscored.

### Supplementary Information

**Additional file 1: S-Figure 1. **Procedure for Withdrawing or Withholding Life-Sustaining Treatment. 

**Additional file 2: S-Table 1.** Subgroup analysis of health care expenditure and covariates, according to Life-Sustaining Treatment decision^a^. **S-Table 2. **Subgroup analysis of health care utilization and covariates, according to Life-Sustaining Treatment decision^a^. 

## Data Availability

All datasets were available from the National Health Insurance Service (NHIS) database that is available upon request after review of the NHIS processes.
