# Supplementary figures and images for: Effects of life-sustaining treatment plans on healthcare expenditure and healthcare utilization
Source: BMC Health Serv Res. 2023 Nov 10;23:1236. doi: 10.1186/s12913-023-10235-x (PMC10638738; doi:10.1186/s12913-023-10235-x)

**Figure legends**


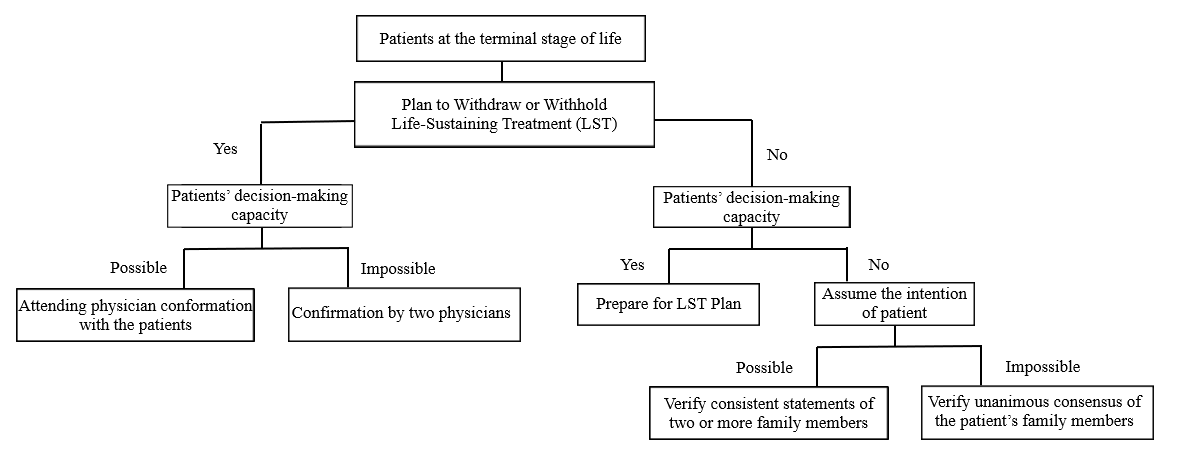


**S-Figure 1. Procedure for Withdrawing or Withholding Life-Sustaining Treatment**

Supplement: Supplementary file 1 — Additional file 1: S-Figure 1. Procedure for Withdrawing or Withholding Life-Sustaining Treatment. [file 12913_2023_10235_MOESM1_ESM.docx]
