# Supplementary material for: Effects of life-sustaining treatment plans on healthcare expenditure and healthcare utilization
Source: BMC Health Serv Res. 2023 Nov 10;23:1236. doi: 10.1186/s12913-023-10235-x (PMC10638738; doi:10.1186/s12913-023-10235-x)
Supplement: Supplementary file 2 — Additional file 2: S-Table 1. Subgroup analysis of health care expenditure and covariates, according to Life-Sustaining Treatment decisiona. S-Table 2. Subgroup analysis of health care utilization and covariates, according to Life-Sustaining Treatment decisiona. [file 12913_2023_10235_MOESM2_ESM.docx]

**S-Table 1. Subgroup analysis of health care expenditure and covariates, according to Life-Sustaining Treatment decision ^a^**

|  |  | **Life-Sustaining Treatment** | | | | | | | | | | | | | | | | | | | | |  |
| --- | --- | --- | --- | --- | --- | --- | --- | --- | --- | --- | --- | --- | --- | --- | --- | --- | --- | --- | --- | --- | --- | --- | --- |
|  |  | Continuation |  | Withdrawal / Withholding | | | | | | | | | | | | | | | | | | |  |
|  |  |  |  | **Total cost** | | | |  | **Hospitalization cost** | | | |  | **Outpatient cost** | | | |  | **Medication cost** | | | |  |
|  |  | **EXP(ß)** |  | **EXP(ß)** | 95% CI | | |  | **EXP(ß)** | 95% CI | | |  | **EXP(ß)** | 95% CI | | |  | **EXP(ß)** | 95% CI | | |  |
|  |  |  |  |  | Lower |  | Upper |  |  | Lower |  | Upper |  |  | Lower |  | Upper |  |  | Lower |  | Upper | |
| **Main illness** | |  |  |  |  |  |  |  |  |  |  |  |  |  |  |  |  |  |  |  |  |  | |
|  | Cancer | 1.00 |  | 0.92 | **(0.89** | - | 0.96) |  | 0.95 | **(0.92** | - | 0.99) |  | 0.70 | **(0.63** | - | 0.78) |  | 0.92 | **(0.81** | - | 1.05) | |
|  | Circulatory system | 1.00 |  | 0.88 | **(0.75** | - | 1.04) |  | 0.91 | **(0.77** | - | 1.07) |  | 0.37 | **(0.25** | - | 0.55) |  | 0.53 | **(0.30** | - | 0.94) | |
|  | Respiratory system | 1.00 |  | 0.90 | **(0.81** | - | 1.01) |  | 0.92 | **(0.82** | - | 1.02) |  | 0.78 | **(0.58** | - | 1.06) |  | 0.82 | **(0.57** | - | 1.19) | |
|  | Others | 1.00 |  | 0.87 | **(0.80** | - | 0.95) |  | 0.90 | **(0.83** | - | 0.97) |  | 0.56 | **(0.44** | - | 0.71) |  | 0.69 | **(0.52** | - | 0.92) | |
| **Sex** |  |  |  |  |  |  |  |  |  |  |  |  |  |  |  |  |  |  |  |  |  |  | |
|  | Male | 1.00 |  | 0.90 | **(0.87** | - | 0.94) |  | 0.93 | **(0.90** | - | 0.97) |  | 0.63 | **(0.57** | - | 0.71) |  | 0.77 | **(0.67** | - | 0.89) | |
|  | Female | 1.00 |  | 0.93 | **(0.88** | - | 0.98) |  | 0.95 | **(0.90** | - | 1.00) |  | 0.70 | **(0.61** | - | 0.80) |  | 1.00 | **(0.83** | - | 1.19) | |
| **Age (years)** | |  |  |  |  |  |  |  |  |  |  |  |  |  |  |  |  |  |  |  |  |  | |
|  | < 50 | 1.00 |  | 1.03 | **(0.91** | - | 1.17) |  | 1.04 | **(0.92** | - | 1.17) |  | 0.78 | **(0.56** | - | 1.10) |  | 1.71 | **(0.93** | - | 3.14) | |
|  | 50-60 | 1.00 |  | 0.93 | **(0.85** | - | 1.01) |  | 0.95 | **(0.87** | - | 1.03) |  | 0.78 | **(0.61** | - | 0.98) |  | 0.99 | **(0.73** | - | 1.35) | |
|  | 60-70 | 1.00 |  | 0.93 | **(0.87** | - | 1.00) |  | 0.96 | **(0.90** | - | 1.03) |  | 0.76 | **(0.63** | - | 0.92) |  | 0.71 | **(0.57** | - | 0.89) | |
|  | 70-80 | 1.00 |  | 0.89 | **(0.84** | - | 0.94) |  | 0.92 | **(0.87** | - | 0.97) |  | 0.63 | **(0.54** | - | 0.75) |  | 0.78 | **(0.63** | - | 0.96) | |
|  | ≥ 80 | 1.00 |  | 0.89 | **(0.83** | - | 0.95) |  | 0.92 | **(0.86** | - | 0.98) |  | 0.50 | **(0.42** | - | 0.60) |  | 0.75 | **(0.61** | - | 0.91) | |
| **Insurance** | |  |  |  |  |  |  |  |  |  |  |  |  |  |  |  |  |  |  |  |  |  | |
|  | Medical aid | 1.00 |  | 0.80 | **(0.72** | - | 0.88) |  | 0.81 | **(0.73** | - | 0.90) |  | 0.67 | **(0.50** | - | 0.90) |  | 0.96 | **(0.69** | - | 1.33) | |
|  | 1 (Low) | 1.00 |  | 0.88 | **(0.81** | - | 0.95) |  | 0.90 | **(0.83** | - | 0.98) |  | 0.60 | **(0.48** | - | 0.75) |  | 0.67 | **(0.50** | - | 0.89) | |
|  | 2 | 1.00 |  | 0.99 | **(0.91** | - | 1.09) |  | 1.00 | **(0.91** | - | 1.10) |  | 0.97 | **(0.74** | - | 1.27) |  | 1.00 | **(0.68** | - | 1.47) | |
|  | 3 | 1.00 |  | 0.90 | **(0.83** |  | 0.98) |  | 0.93 | **(0.85** |  | 1.00) |  | 0.72 | **(0.57** |  | 0.91) |  | 0.69 | **(0.52** |  | 0.91) | |
|  | 4 | 1.00 |  | 0.91 | **(0.84** | - | 0.98) |  | 0.92 | **(0.86** | - | 0.99) |  | 0.76 | **(0.63** | - | 0.93) |  | 1.27 | **(0.99** | - | 1.63) | |
|  | 5 (High) | 1.00 |  | 0.96 | **(0.90** | - | 1.01) |  | 1.00 | **(0.94** | - | 1.06) |  | 0.55 | **(0.47** | - | 0.64) |  | 0.62 | **(0.51** | - | 0.76) | |
| **Medical institution** | | |  |  |  |  |  |  |  |  |  |  |  |  |  |  |  |  |  |  |  |  | |
|  | Tertiary hospital | 1.00 |  | 0.93 | **(0.90** | - | 0.97) |  | 0.96 | **(0.93** | - | 1.00) |  | 0.61 | **(0.55** | - | 0.68) |  | 0.93 | **(0.81** | - | 1.07) | |
|  | General hospital | 1.00 |  | 0.86 | **(0.81** | - | 0.91) |  | 0.87 | **(0.82** | - | 0.93) |  | 0.83 | **(0.71** | - | 0.97) |  | 0.67 | **(0.55** | - | 0.82) | |
|  | Others | 1.00 |  | 1.53 | (0.51 | - | 4.55) |  | 1.53 | (0.50 |  | 4.68) |  | **–** |  |  |  |  | **–** |  |  |  | |
|  |  |  |  |  |  |  |  |  |  |  |  |  |  |  |  |  |  |  |  |  |  |  | |
| ^a^ The reference for the costs (total, hospitalization, outpatient, medication) was the LST Continuation group (EXP(ß) = 1.00). | | | | | | | | | | | | | | | | | | | | | | |  |

**S-Table 2. Subgroup analysis of health care utilization and covariates, according to Life-Sustaining Treatment decision ^a^**

|  |  | **Life-Sustaining Treatment** | | | | | | | | | | |  |  |  |
| --- | --- | --- | --- | --- | --- | --- | --- | --- | --- | --- | --- | --- | --- | --- | --- |
|  |  | **Continuation** |  | **Withdrawal / Withholding** | | | | | | | | |  |  |  |
|  |  |  |  | **Days of hospitalization** | | | |  | **Outpatient visit** | | | |  |  |  |
|  |  | **EXP(ß)** |  | **EXP(ß)** | **95% CI** | | |  | **EXP(ß)** | **95% CI** | | |  |  |  |
|  |  |  |  |  | **Lower** |  | **Upper** |  |  | **Lower** |  | **Upper** |  |  |  |
| **Main illness** | |  |  |  |  |  |  |  |  |  |  |  |  |  |  |
|  | Cancer | 1.00 |  | 0.96 | (0.93 | - | 0.99) |  | 0.90 | (0.87 | - | 0.93) |  |  |  |
|  | Circulatory system | 1.00 |  | 0.80 | (0.64 | - | 0.99) |  | 0.96 | (0.79 | - | 1.17) |  |  |  |
|  | Respiratory system | 1.00 |  | 0.84 | (0.74 | - | 0.95) |  | 1.09 | (0.96 | - | 1.25) |  |  |  |
|  | Others | 1.00 |  | 0.84 | (0.77 | - | 0.93) |  | 0.93 | (0.84 | - | 1.03) |  |  |  |
| **Sex** | |  |  |  |  |  |  |  |  |  |  |  |  |  |  |
|  | Male | 1.00 |  | 0.92 | (0.89 | - | 0.96) |  | 0.92 | (0.88 | - | 0.96) |  |  |  |
|  | Female | 1.00 |  | 0.91 | (0.86 | - | 0.96) |  | 0.93 | (0.88 | - | 0.99) |  |  |  |
| **Age (years)** | | |  |  |  |  |  |  |  |  |  |  |  |  |  |
|  | < 50 | 1.00 |  | 1.00 | (0.90 | - | 1.11) |  | 0.99 | (0.88 | - | 1.12) |  |  |  |
|  | 50-60 | 1.00 |  | 0.99 | (0.92 | - | 1.07) |  | 0.92 | (0.85 | - | 1.00) |  |  |  |
|  | 60-70 | 1.00 |  | 0.93 | (0.87 | - | 0.99) |  | 0.90 | (0.85 | - | 0.96) |  |  |  |
|  | 70-80 | 1.00 |  | 0.92 | (0.86 | - | 0.97) |  | 0.89 | (0.84 | - | 0.95) |  |  |  |
|  | ≥ 80 | 1.00 |  | 0.86 | (0.80 | - | 0.92) |  | 0.97 | (0.90 | - | 1.04) |  |  |  |
| **Insurance** | |  |  |  |  |  |  |  |  |  |  |  |  |  |  |
|  | Medical aid | 1.00 |  | 0.91 | (0.81 | - | 1.02) |  | 0.91 | (0.81 | - | 1.03) |  |  |  |
|  | 1 (Low) | 1.00 |  | 0.94 | (0.86 | - | 1.03) |  | 0.90 | (0.82 | - | 0.98) |  |  |  |
|  | 2 | 1.00 |  | 0.93 | (0.84 | - | 1.02) |  | 0.87 | (0.79 | - | 0.96) |  |  |  |
|  | 3 | 1.00 |  | 0.91 | (0.84 |  | 0.99) |  | 0.95 | (0.87 |  | 1.03) |  |  |  |
|  | 4 | 1.00 |  | 0.91 | (0.85 | - | 0.98) |  | 0.92 | (0.86 | - | 0.99) |  |  |  |
|  | 5 (High) | 1.00 |  | 0.92 | (0.87 | - | 0.97) |  | 0.94 | (0.89 | - | 1.00) |  |  |  |
| **Medical institution** | |  |  |  |  |  |  |  |  |  |  |  |  |  |  |
|  | Tertiary hospital | 1.00 |  | 0.92 | (0.89 | - | 0.96) |  | 0.90 | (0.87 | - | 0.94) |  |  |  |
|  | General hospital | 1.00 |  | 0.91 | (0.85 | - | 0.96) |  | 0.97 | (0.91 | - | 1.04) |  |  |  |
|  | Others | 1.00 |  | 0.88 | (0.27 | - | 2.85) |  | 0.63 | (0.24 |  | 1.65) |  |  |  |
| ^a^ The reference for the costs (total, hospitalization, outpatient, medication) was the LST Continuation group (EXP(ß) = 1.00). | | | | | | | | | | | | | | | |
